# Infertility etiologies are genetically and clinically linked with other diseases in single meta-diseases

**DOI:** 10.1186/s12958-015-0029-9

**Published:** 2015-04-15

**Authors:** Juan J Tarín, Miguel A García-Pérez, Toshio Hamatani, Antonio Cano

**Affiliations:** Department of Functional Biology and Physical Anthropology, Faculty of Biological Sciences, University of Valencia, Burjassot, Valencia 46100 Spain; Department of Genetics, Faculty of Biological Sciences, University of Valencia, Burjassot, Valencia 46100 Spain; Research Unit-INCLIVA, Hospital Clínico de Valencia, Valencia, 46010 Spain; Department of Obstetrics and Gynecology, Keio University School of Medicine, Tokyo, 160-8582 Japan; Department of Pediatrics, Obstetrics and Gynecology, Faculty of Medicine, University of Valencia, Valencia, 46010 Spain; Service of Obstetrics and Gynecology, University Clinic Hospital, Valencia, 46010 Spain

**Keywords:** Cancer, Disease, Gene expression, Molecular pathways, Phenomics

## Abstract

The present review aims to ascertain whether different infertility etiologies share particular genes and/or molecular pathways with other pathologies and are associated with distinct and particular risks of later-life morbidity and mortality. In order to reach this aim, we use two different sources of information: (1) a public web server named DiseaseConnect (http://disease-connect.org) focused on the analysis of common genes and molecular mechanisms shared by diseases by integrating comprehensive omics and literature data; and (2) a literature search directed to find clinical comorbid relationships of infertility etiologies with only those diseases appearing after infertility is manifested. This literature search is performed because DiseaseConnect web server does not discriminate between pathologies emerging before, concomitantly or after infertility is manifested. Data show that different infertility etiologies not only share particular genes and/or molecular pathways with other pathologies but they have distinct clinical relationships with other diseases appearing after infertility is manifested. In particular, (1) testicular and high-grade prostate cancer in male infertility; (2) non-fatal stroke and endometrial cancer, and likely non-fatal coronary heart disease and ovarian cancer in polycystic ovary syndrome; (3) osteoporosis, psychosexual dysfunction, mood disorders and dementia in premature ovarian failure; (4) breast and ovarian cancer in carriers of BRCA1/2 mutations in diminished ovarian reserve; (5) clear cell and endometrioid histologic subtypes of invasive ovarian cancer, and likely low-grade serous invasive ovarian cancer, melanoma and non-Hodgkin lymphoma in endometriosis; and (6) endometrial and ovarian cancer in idiopathic infertility. The present data endorse the principle that the occurrence of a disease (in our case infertility) is non-random in the population and suggest that different infertility etiologies are genetically and clinically linked with other diseases in single meta-diseases. This finding opens new insights for clinicians and reproductive biologists to treat infertility problems using a phenomic approach instead of considering infertility as an isolated and exclusive disease of the reproductive system/hypothalamic–pituitary–gonadal axis. In agreement with a previous validation analysis of the utility of DiseaseConnect web server, the present study does not show a univocal correspondence between common gene expression and clinical comorbid relationship. Further work is needed to untangle the potential genetic, epigenetic and phenotypic relationships that may be present among different infertility etiologies, morbid conditions and physical/cognitive traits.

## Background

Infertility [Unified Medical Language System (UMLS) identification (ID): C0021359] is the inability to conceive for at least one year after trying and having unprotected sex [1]. It is estimated that about 15% of couples are infertile [2]. Male and female infertility account for about a third of infertility cases, respectively, whereas the other third of cases is because of either problems in both partners or unknown reasons [1].

Between 15-30% of infertile men and ≈ 10% of infertile women display genetic abnormalities, including chromosome aberrations, single- or multiple-gene mutations, and polymorphisms (for review, see [3]). Infertile men and women may also display mitochondrial and epigenetic disturbances, although the incidence of these defects has not been yet established (for review, see [3]). In addition to these genetic/epigenetic backgrounds, many infertile subjects are exposed to environmental toxicants/endocrine disruptors and/or display immunological and/or endogenous hormonal disturbances that not only may jeopardize reproductive health but also later-life morbidity and mortality.

Recently, a public web server (http://disease-connect.org) named DiseaseConnect has been created [4,5]. This server is focused on the analysis of common genes and molecular mechanisms shared by diseases by integrating comprehensive omics and literature data. This is a novel approach that aims to search common pathogenic mechanisms that link diseases together in a single meta-disease instead of the traditional approach of splitting the phenome (i.e., the set of phenotypes attributable to sequence variation in the human genome and specific environmental influences) into discrete entities called diseases [6].

Noteworthy, the utility of DiseaseConnect server for biologists to study mechanism-based disease connectivity was validated by Liu et al. [4]. In particular, these authors showed that diseases with shared molecular mechanisms are “likely” to be linked with clinical comorbidity data and to have the same drug treatment. However, as this validation was based on probabilistic grounds, a univocal correspondence between common gene expression and clinical comorbid relationship cannot be expected. In other words, common gene expression associations reported by DiseaseConnect web server [5] do not necessarily imply the presence of clinical comorbid relationships. In addition, clinical comorbid relationships may appear or not simultaneously in life. For instance, diseases typically characteristic of older post-reproductive adults such as senile dementia and Alzheimer's disease manifest much later in life than infertility. In contrast, diabetes mellitus or urinary tract infection may manifest concomitantly or even before infertility is evidenced. Thus, it is not possible to discriminate from DiseaseConnect clinical comorbid relationships whether infertility is linked with other diseases because it shares genes and molecular pathways with those diseases or because it results from the morbid effects of other pre-existing pathological conditions. Moreover, infertility is a heterogeneous condition with multiple genetic, epigenetic, mitochondrial, immunological, hormonal and environmental etiologies [3]. Therefore, it is not possible to infer that all the infertility etiologies share the same abnormalities in gene expression and/or infertility-linked pathologies. Instead, we may expect that each particular cause of infertility has its own genetic and/or comorbid relationships with other diseases.

The present review aims to ascertain whether different infertility etiologies share particular genes and/or molecular pathways with other pathologies and are associated with distinct and particular risks of later-life morbidity and mortality. In order to reach this aim, we use two different sources of information: (1) DiseaseConnect web server for the analysis of common genes and molecular mechanisms shared by diseases; and (2) a literature search focused on clinical comorbid relationships of infertility etiologies with only those diseases appearing after infertility is manifested. We perform this literature search because DiseaseConnect web server [5] does not discriminate between pathologies emerging before, concomitantly or after infertility is manifested.

## Methods

As mentioned above, this study used data on common genes and molecular mechanisms shared by infertility etiologies and other diseases from DiseaseConnect web server [5]. In addition, we performed a literature search aimed to find clinical comorbid relationships between infertility etiologies and other diseases appearing later in life, based on publications up to December 2014 identified by PubMed database using the following key words: polycystic ovary syndrome (PCOS), premature ovarian failure (POF), diminished ovarian reserve (DOR), endometriosis, fertility drugs, idiopathic/unexplained/unknown infertility, cancer, cardiovascular disease, morbidity, mortality, semen quality, oligozoospermia, teratozoospermia, asthenozoospermia and normozoospermia. We also carried out a manual search to explore the references cited in the primary articles.

The PubMed and manual literature search showed that the majority of studies did not determine whether cancer risk is associated with infertility itself or with the exposure of infertile women to fertility drugs. Notwithstanding, recent non-systematic reviews [7], systematic reviews [8,9], and systematic reviews and meta-analyses [10-12] suggest that the use of fertility drugs does not increase the risk of breast, ovarian, endometrial and cervical cancer. In contrast, some studies report increased risks of borderline (low malignant potential) ovarian tumors in women treated with fertility drugs for in-vitro fertilization (IVF) (for systematic reviews, see [8,9]). These findings, however, are likely a consequence of surveillance bias and younger age of subfertile women (for systematic review, see [8]). Thus, the association between causes of infertility and breast-gynecological cancers reported in the present review can be attributed to infertility etiology in itself, not to the use of fertility drugs.

Hypergeometric test was applied by DiseaseConnect [5] to assess the significance of the Genome-Wide Association (GWAS), Online Mendelian Inheritance in Man (OMIM) and differentially expressed genes (DEG) genes shared by specific infertility etiologies and particular diseases. Hypergeometric test calculates the statistical significance of having drawn k successes (out of n total draws, without replacement, wherein each draw is either a success or a failure) from a finite population of size N containing exactly K successes. Tables 1, 2, 3 and 4 show the hypergeometric P-values that quantify the strength of the disease-disease connection in the number of shared genes.

**Table 1:** **Relation of male infertility** (**UMLS ID: C0021364) with other diseases that significantly share GWAS/OMIM/DEG genes** [5]

**Diseases**	**P value** ^**a**^
Infertility	<0.0001
Mutation abnormality	0.0004
Malignant neoplasm of urinary organ, unspecified	0.0010
Bladder neoplasm	0.0010
Urologic neoplasms	0.0010
Carcinoma of bladder	0.0010
Neoplasm of unspecified nature of bladder	0.0010
Urinary bladder diseases	0.0011
B cell lymphomas	0.0029
Liposarcoma, myxoid	0.0036
Liposarcoma	0.0037
Neoplasms, fibrous tissue	0.0042
Juvenile dermatomyositis	0.0044
Amyopathic dermatomyositis	0.0044
Fibrosarcoma	0.0044
Neoplasms, connective tissue	0.0050
Liposarcoma, dedifferentiated	0.0058
Frontotemporal lobar degeneration	0.0074
Lymphoma, non Hodgkin	0.0077
TDP 43 proteinopathies	0.0098
Myositis	0.0099

^a^A hypergeometric test was applied by DiseaseConnect [5] to assess the significance of the GWAS/OMIM/DEG genes shared by male infertility and a particular disease.

**Table 2:** **Relation of PCOS** (**UMLS ID: C0032460) with other diseases that significantly share GWAS/OMIM/DEG genes** [5]

**Diseases**	**P value** ^**a**^
Ovarian cysts	<0.0001
Gonadal dysgenesis	0.0002
Leydig cell hypoplasia, type II	0.0015
Donohue syndrome	0.0015
Sulfocysteinuria	0.0015
Primary hypogonadism	0.0015
Diabetes mellitus, insulin resistant, with acanthosis nigricans	0.0015
Sulfite oxidase deficiency	0.0015
Ovarian hyperstimulation syndrome	0.0015
Rabson Mendenhall syndrome	0.0015
Eunuchism	0.0030
Pseudohermaphroditism	0.0030
ACTH deficiency, isolated	0.0030
Isolated lutropin deficiency (disorder)	0.0030
Lassa fever	0.0031
Arenaviridae infections	0.0031
Hemorrhagic fevers, viral	0.0032
Precocious puberty	0.0046
Liposarcoma, dedifferentiated	0.0057
Gonadal dysgenesis, 46,XX	0.0061
46, XX disorders of sex development	0.0061
Synovial sarcoma	0.0067
Multiple system congenital anomalies not elsewhere classified (NEC) in Systematized Nomenclature of Medicine Clinical Terms (SNOMED-CT)	0.0076

^a^A hypergeometric test was applied by DiseaseConnect [5] to assess the significance of the GWAS/OMIM/DEG genes shared by PCOS and a particular disease.

**Table 3:** **Relation of POF** (**UMLS ID: C0085215) with other diseases that significantly share GWAS/OMIM/DEG genes** [5]

**Diseases**	**P value** ^**a**^
Hypergonadotropic ovarian failure, X linked	<0.0001
Premature menopause	<0.0001
Fragile X tremor/ataxia syndrome	<0.0001
POF7 (disorder)	<0.0001
Spermatogenic failure 8	<0.0001
Hypoaldosteronism	<0.0001
Sex chromosome disorders	<0.0001
Adrenal cortical hypofunction	<0.0001
Fragile X syndrome	<0.0001
Adrenal gland hypofunction	0.0001
Adrenal gland diseases	0.0005
POF5	0.0008
POF6	0.0008
POF2b	0.0008
Sex chromosome aberrations	0.0008
Ptosis	0.0016
Uterine prolapse without mention of vaginal wall prolapse	0.0016
Eyelid diseases	0.0024
Blepharophimosis	0.0040
Blepharoptosis	0.0071

^a^A hypergeometric test was applied by DiseaseConnect [5] to assess the significance of the GWAS/OMIM/DEG genes shared by POF and a particular disease.

**Table 4:** **Relation of endometriosis** (**UMLS ID: C0014175) with other diseases that significantly share GWAS/OMIM/DEG genes** [5]

**Diseases**	**P value** ^**a**^
Female genital diseases	<0.0001
Uterine diseases	<0.0001
Myocardial infarction	<0.0001
Coronary artery disease	<0.0001
Sleep disorders, intrinsic	<0.0001
Parasomnia	<0.0001
Myocardial ischemia	<0.0001
Dementia due to specified medical condition	<0.0001
Restless legs syndrome	0.0002
Diabetes mellitus, non-insulin dependent	0.0005
Sex reversal, female, with dysgenesis of kidneys, adrenals, and lungs	0.0016
46,XX testicular disorders of sex development	0.0016
Disorder of pancreatic internal secretion	0.0017
Hyperandrogenism	0.0032
HIV infections	0.0044
Carotid atherosclerosis	0.0063
Pancreatic intraductal papillary mucinous adenoma	0.0074
Aortic eneurysm, abdominal	0.0095

^a^A hypergeometric test was applied by DiseaseConnect [5] to assess the significance of the GWAS/OMIM/DEG genes shared by endometriosis and a particular disease.

## Genetic and clinical associations of male infertility with other diseases

Male infertility (UMLS ID: C0021364) is a term used if a man has not been able to get a woman pregnant after at least one year of trying [1]. In the context of assisted reproductive technology (ART), the Centers for Disease Control and Prevention [13] define male factor as any cause of male infertility due to low sperm count or problems with sperm function that makes it difficult for a sperm to fertilize an oocyte under normal conditions.

Male infertility (UMLS ID: C0021364) shares genes with other pathologies (Table 1). To date, a total of 168 GWAS/OMIM/DEG genes have been found to be significantly associated with male infertility [5]. These genes are involved in ribosome, pancreatic cancer and proteasome pathways [Kyoto Encyclopedia of Genes and Genomes (KEGG) ID: hsa03010, hsa05212 and hsa03050, respectively]. These pathways play an important role in metabolism, genetic information processing (DNA replication, repair and transcription, RNA degradation and translation, and protein folding, sorting and degradation), environmental information processing, cellular processes and human diseases such as cancer, drug resistance, substance dependence, and endocrine, metabolic, cardiovascular, immune, infectious (bacterial, viral and parasitic) and degenerative diseases [14]. Not surprisingly, male infertility is associated with increased risk of testicular (for review, see [15] and for systematic review, see [16]) and high-grade prostate (for review, see [17]) cancer. In addition, men undergoing an infertility evaluation with two or more semen abnormalities (including semen volume, concentration and motility) have higher risks of death than men with normal semen parameters after controlling for baseline comorbidity [18]. The risk adjustment for baseline comorbidity performed by Eisenberg et al. [18] deserves special mention. Actually, the presence of medical disorders such as obesity (and underweight), diabetes mellitus, metabolic syndrome, hypertension and bacterial/viral infections not only may impair semen quality/production [19] (for review, see [20] and for systematic reviews and meta-analyses, see [21,22]) but also may increase morbidity and/or mortality in subsequent years. Furthermore, deficiencies in certain nutrients such as vitamins C and E, folate, selenium and zinc, and lifestyle habits including substance abuse (e.g., alcohol, cocaine and cannabis) and smoking may reduce sperm quality (for review, see [20] and for systematic review and meta-analysis, see [23]) and life expectancy (for reviews, see [24-27]). Notwithstanding, recent studies have found no significant effect of smoking and alcohol consumption on motile sperm concentration [28] and sperm morphology [29]. In addition, the use of cannabis seems to affect only sperm morphology [29].

## Genetic and clinical associations of female infertility etiologies with other diseases

Female infertility (UMLS ID: C0021361) is a term used when a woman has not been able to get pregnant after at least one year of trying [1]. In the context of ART, female infertility is split into several etiologies including tubal factor, ovulatory dysfunction, diminished ovarian reserve, endometriosis and uterine factor [13]. Likewise, DiseaseConnect web server [5] segregates female infertility into several etiologies including PCOS, POF and endometriosis (see below). In contrast, literature provides studies that analyze the association of “overall” female infertility with later-life morbidity and mortality without taking into account infertility etiologies. The majority of these studies report increased risks of ovarian cancer in infertile women, especially when they remain nulliparous (for reviews, see [9,30]). Moreover, childless women that eventually have a child after ≥ 5 years of self-reported infertility have higher risks of cardiovascular events (hospitalization or death caused by coronary heart disease, stroke or heart failure) during the subsequent 11.9 years after giving birth than women reporting no history of infertility that conceive within 12 months of trying [31].

In addition, mortality risk for a woman whereas it is pregnant or within 42 days of termination of pregnancy is higher in IVF pregnancies than in the general population [32,33]. This is most likely to be associated with the advanced reproductive age and higher incidence of multiple pregnancy and Cesarean section exhibited by IVF women [33]. Moreover, IVF women display a higher prevalence of hypertension and likely stroke during the following 8.6 years after delivery compared with primiparous control mothers from the general population [34]. However, other authors [32,33] have reported that all-cause mortality risks of treated and non-treated IVF women are significantly lower than those of the general female population of the same age. Similarly, women who give birth after fertility therapy have fewer cardiovascular events, all-cause mortality, subsequent depression, alcoholism, and self-harm than control women from the general population who deliver without receiving fertility therapy [35]. These findings may be explained by the healthier condition/behavior and/or higher socio-economic status of infertile women compared with their general population counterparts [30,35,36].

### PCOS

PCOS (UMLS ID: C0032460) is a complex and heterogeneous disease influenced by many environmental and genetic factors (for review, see [37]). It accounts for 90-95% of infertile women suffering from ovulatory dysfunction attending infertility clinics in U.S. (for review, see [38]). According to the Rotterdam criteria [39], PCOS is diagnosed if a woman meets any two of the following three criteria: (1) oligo- and/or anovulation; (2) clinical and/or biochemical signs of hyperandrogenism; and (3) polycystic ovaries on ultrasounds and exclusion of other known disorders of hyperandrogenemia (congenital adrenal hyperplasias, androgen-secreting tumors and Cushing’s syndrome).

DiseaseConnect web server [5] reports no molecular pathways associated with this condition. However, 52 protein complexes are significantly linked with the 23 GWAS/OMIM/DEG genes related with PCOS [5]. Twenty four of these 52 complexes are members of the ubiquitin E3 ligase family. These complexes are involved in protein deubiquitination and ubiquitination, proteasomal ubiquitin-dependent protein catabolism, protein amino acid autophosphorylation, dephosphorylation and phosphorylation, response to DNA damage stimulus, mitotic cell cycle and cell aging [40]. In addition, PCOS shares genes with other pathologies including gonadal dysgenesis, precocious puberty and type 2 diabetes mellitus (Table 2).

Epidemiological studies show that PCOS is associated with increased risks of insulin resistance, impaired glucose tolerance, gestational diabetes, type 2 diabetes mellitus and metabolic syndrome (cited by [41]). Consequently, PCOS is linked with higher risk of non-fatal stroke and possibly non-fatal coronary heart disease (for systematic review and meta-analysis, see [42]).

As genetic factors play an important role in the etiology of PCOS with an estimated heritability of 65% [43], not surprisingly several studies [44,45] report that mothers, fathers and brothers of women with PCOS are more likely to have cardiovascular disease in later adult life. These studies, however, did not find a significant relationship between PCOS women and a family history of diabetes. On the contrary, Louwers et al. [46] evidenced that mothers (but not fathers) above age 60 years of patients with PCOS display increased all-cause mortality, most likely as result of type 2 diabetes mellitus.

It appears that the prolonged exposure to high levels of estrogen associated with progesterone deficiency/resistance in PCOS women [47] may induce hyperplasia and uncontrolled differentiation of the endometrium. Accordingly, the probability of random mutation and DNA replication errors leading to development of cancer may be increased. In fact, PCOS is associated with higher risk of endometrial and likely ovarian cancer although not breast cancer (for review, see [48], for systematic review, see [9] and for systematic reviews and meta-analyses, see [49,50]). Moreover, the insulin resistance and hyperinsulinemia exhibited by PCOS women may lead to excessive secretion of androgens, luteinizing hormone (LH) and insulin-like growth factor I (IGF-I) which are additional risk factors for endometrial and ovarian cancer (for reviews, see [51,52]). Noteworthy, among the various PCOS phenotypes, those displaying hyperandrogenism have the highest metabolic morbidity and prevalence of cardiovascular risk factors including obesity/overweight, hypertension, insulin resistance, dyslipidemia and metabolic syndrome [41,53] (for review, see [54]).

### POF

POF (UMLS ID: C0085215) is a condition characterized by sustained (4 months or more) amenorrhea, elevated early-follicular levels of follicle stimulating hormone (FSH) and low estrogen levels in women less than 40 years of age (for review, see [55]). In contrast to natural menopause, women diagnosed with POF may undergo unpredictable ovarian function leading to intermittent and unpredictable menses in 50% of cases, and conceive and deliver a child in ≈ 5 to 10% of cases. For this reason, other authors redefine this condition as 4 months or more of disordered menses (amenorrhea, oligomenorrhea, polymenorrhea, or metrorrhagia), and use the term primary ovarian insufficiency (POI) instead of POF (for review, see [56]).

POF is a multifactorial condition including multiple causes such as idiopathic, X-chromosome alterations, autosomal genetic disorders, autoimmune, infections and iatrogenic following treatment with immunosuppressant drugs, surgery for gynecological disorders or chemotherapy/pelvic irradiation for malignancy (for reviews, see [55,57,58]). The incidence of POF is estimated to be 1% in women under the age of 40, 0.1% under 30 and 0.01% under 20. Nevertheless, the prevalence of POF is continuously increasing due to the higher survival following malignant disease (for review, see [57]).

Like in PCOS, no molecular pathways associated with POF have been reported so far by DiseaseConnect web server [5]. Only the protein complex, leukemia inhibitory factor (LIF) receptor (LIFR)-LIF-glycoprotein 130 (gp130), which is involved in interleukin receptor signaling pathway and differentiation of the nervous system [40], is significantly linked with the 12 GWAS/OMIM/DEG genes of POF reported by DiseaseConnect [5]. Moreover, POF shares genes with other pathologies such as X-linked hypergonadotropic ovarian failure, adrenal gland diseases and uterine prolapse (Table 3).

Epidemiological studies show that POF women have increased risk of osteoporosis, psychosexual dysfunction, mood disorders and dementia as well as raised risk for all-cause mortality including cancer, cardiovascular disease and other causes (e.g., neurological, respiratory, renal, liver disease, diabetes mellitus, etc.). On the contrary, it is associated with lower risk of breast and endometrial cancer due to the reduction in levels of endogenous estrogens ([59,60] and references quoted therein; for reviews, see [57,61]).

It is thought that some changes in the cardiac and vascular structure and physiology that take place in menopause are due to the reduced levels of estrogen and relatively increased serum androgens associated with this condition (for review, see [62]). The early and prolonged loss of ovarian 17β-estradiol (E_2_) in POF women may lead to a doubled lifetime risk for dementia and a five-fold increase risk of mortality from neurological disorders (for review, see [63]). In addition, low E_2_ levels are involved in the typical loss of bone calcium and osteoporosis, although the concurrent increase in circulating FSH (i.e., the increased ratio of FSH to E_2_) is likely the main responsible for bone resorption (for review, see [64]). Likewise, an increase in the ratio between mitogenic [gonadotropin-releasing hormone (GnRH) and gonadotropins] and differentiative (sex steroids) hormones may lead to aberrant cell proliferation and cancer (for review, see [64]).

### DOR

DOR is another cause of infertility that shares many features and etiologies with POF/POI. It is defined as a decreased number and/or quality of oocytes (for review, see [65]), or when the ability of the ovary to produce oocytes is reduced because of congenital, medical or surgical causes, or advanced age [13]. In women less than 35 years of age, DOR is the cause of infertility that contributes more than others to the relatively low success percentages observed in ART cycles, especially in fresh autologous cycles. In particular, the 2012 national clinic summary report by the Society for Assisted Reproductive Technology (SART) [66] shows that the live-birth percentage per fresh autologous cycle in DOR women is only 30.3% (542/1789) versus 36.6% (1135/3102) in multiple female factors, 37.2% (929/2498) in other factors, 38.3% (988/6580) in female and male factors, 40.2% (1116/2776) in tubal factor, 41.2% (184/447) in uterine factor, 41.8% (813/1946) in endometriosis, 42.1% (1796/4265) in ovulatory dysfunction, 42.4% (2358/5562) in unknown factor, and 44.6% (4325/9697) in male factor.

DOR has multiple causes including genetic, environmental, autoimmune, idiopathic, advanced age and iatrogenic (ovarian surgery, chemotherapy and radiation) (for reviews, see [55,65,67]). DOR women present regular menses and alterations of ovarian reserve tests, such as increased early follicular FSH levels, lower anti-Müllerian hormone serum concentrations, decreased antral follicular count, high dehydroepiandrosterone (DHEA)-sulfate (DHEAS) levels and low testosterone concentrations due to DHEA conversion insufficiency, especially in women over 40 years of age [68] (for review, see [55]). The prevalence of DOR in women attending infertility centers in U.S. has been steadily increasing from 10% in 2003 to 17% in 2012 [66] (note that these percentages include women suffering only from DOR. Figures may be higher if women with multiple infertility factors were taken into account).

DiseaseConnect web server [5] does not provide any information about this particular condition. However, epidemiological studies indicate that DOR women ≤ 35 years of age exhibit higher levels of several cardiovascular disease risk markers including insulin resistance, C-reactive protein, triglyceride and high-density lipoprotein compared with women with normal ovarian reserve [69]. A link between DOR and breast and ovarian cancer has been established in carriers of *BRCA1/2* mutations [these genes are members of the ataxia-telangiectasia mutated (ATM)-mediated DNA double-strand break repair family of genes that act via a homologous recombination mechanism]. In fact, women carrying mutations in *BRCA1* exhibit lower ovarian reserve evaluated on the basis of lower serum concentration of anti-Müllerian hormone levels than women with no *BRCA1/2* mutations or carrying *BRCA2* mutations irrespectively of the presence [70] or the absence [71] of a personal history of breast or ovarian cancer. Moreover, *BRCA1*-deficient women have higher risk of breast and ovarian cancer ([72,73] and references quoted therein), low response to exogenous ovarian stimulation [74] and may experience earlier natural menopause [75-77]. Notwithstanding, a recent study has reported that *BRCA1* mutation carriers do not display a higher risk of earlier natural menopause when compared with their non-carrier relatives [78].

According to Oktay et al. [79], the function of DNA repair genes such as *BRCA1* declines with age. This decrease causes accumulation of lethal DNA double-strand breaks in primordial follicles which may activate apoptotic cell death mechanisms in oocytes/follicles to prevent propagation of resultant severe mutations. Such an age-associated decline in DNA-repair function is exacerbated in *BRCA1*-mutant carriers. For this reason, these women display a lower ovarian reserve compared with women without *BRCA1/2* mutations [70]. Of note, the effects on ovarian reserve and risk of breast and ovarian cancer in *BRCA2*-mutant carriers is less pronounced and/or delayed compared with *BRCA1*-mutant carriers because of a much later decrease in *BRCA* function. Nonetheless, women with a *BRCA2* mutation, not only have increased lifetime risks of developing breast and ovarian cancer like *BRCA1*-mutant carriers, they also exhibit a higher incidence of pancreatic cancer compared with the general population ([73] and references quoted therein).

### Endometriosis

Endometriosis (UMLS ID: C0014175) is an estrogen-dependent, inflammatory, chronic disorder characterized by the presence of endometrial-like glands and stroma outside the uterine cavity and musculature, mainly located on the pelvic peritoneum, ovaries and the retrocervical septum (also incorrectly named rectovaginal septum [80]). Nevertheless, it can be found less commonly outside the pelvis at most sites of the body including the lungs, skin, lymph nodes, breast and brain (for reviews, see [81,82]). Women with endometriosis may be asymptomatic or, depending on the site of the lesions, may suffer from dysmenorrhea, pelvic pain (particularly during menstruation), dyspareunia, and/or pain associated with urination or bowel movements (for review, see [83]). Endometriosis is uncommon before menarche and usually regresses after menopause (for review, see [84]). The prevalence of endometriosis is 25-30% among infertile women (for review, see [84]) and up to 90% among reproductive women with chronic pelvic pain (for review, [85]). The etiology of endometriosis is still unknown but it is thought it is influenced by genetic, epigenetic, hormonal, environmental and immunological factors (for reviews, see [81,83,86-88]).

To date, a total of 24 GWAS/OMIM/DEG genes have been reported by DiseaseConnect web server [5] to be significantly associated with this condition. These genes are involved in vitamin B_6_ metabolism pathway (KEGG pathway ID: hsa00750), which plays an important role in metabolism, genetic/environmental information processing, cellular processes and human diseases [7]. Endometriosis shares genes with other pathologies including myocardial infarction, coronary artery disease, sleep disorders, restless legs syndrome and type 2 diabetes mellitus (Table 4).

Endometriosis does not induce a catabolic state, metabolic disturbances or death like a malignancy, but it shares common characteristic of ovarian cancer including invasion, unrestrained growth, angiogenesis, decreased number of cells undergoing apoptosis, and genetic, epigenetic and biochemical alterations (for reviews, see [82,83,85,89,90]). Although the direct progression of endometriosis lesions to ovarian neoplasms has been only rarely demonstrated (for review, see [90]), literature shows a significant association of endometriosis with clear cell and endometrioid histologic subtypes of invasive ovarian cancers, and likely low-grade serous invasive ovarian cancer, melanoma and non-Hodgkin lymphoma (for reviews, see [81,82,85,91-93]).

### Unexplained infertility

Unexplained (also named unknown or idiopathic) cause of infertility (UMLS ID: C0404585) is a diagnostic category used when no cause of infertility is found in either the woman or the man [13]. Despite many researchers and institutions have exerted substantial effort and invested considerable financial resources to improve infertility diagnosis, the incidence of unexplained infertility in couples attending infertility centers in U.S. has been kept more or less constant since 2003 (11-13%) [66].

Like in DOR, DiseaseConnect web server [5] does not include the term unexplained, unknown or idiopathic infertility in its repertoire and, therefore, it does not provide any information on this condition. Nonetheless, epidemiological studies show that, in addition to endometrial cancer [94], this infertility condition is linked to ovarian cancer (for review, see [91]). In particular, serous borderline, serous invasive, endometrioid and clear cell histological subtypes of ovarian cancer [95]. It has been hypothesized that these women may display abnormal regulation of inflammatory responses by prostaglandins [95] or suffer from preexisting cancer [94] or an underlying pathology associated with both reduced fertility and increased cancer risk [96].

## Concluding remarks

This review has found evidence that different infertility etiologies not only share particular genes and/or molecular pathways with other pathologies but they have distinct clinical relationships with other diseases appearing later in life. The present data endorse the principle that the occurrence of a disease (in our case infertility) is non-random in the population [6] and suggest that different infertility etiologies are genetically and clinically linked with other diseases in single meta-diseases. This finding opens new insights for clinicians and reproductive biologists to treat infertility problems using a phenomic approach instead of considering infertility as an isolated and exclusive disease of the reproductive system/hypothalamic–pituitary–gonadal axis.

We should emphasize that we have used two different sources of information to find this evidence: (1) DiseaseConnect web server [5] to search for common genes and molecular mechanisms shared by infertility etiologies and other diseases; and (2) a literature search focused on clinical comorbid relationships of infertility etiologies with only those diseases appearing after infertility is manifested. Of note, the present study does not show a univocal correspondence between common gene expression and clinical comorbid relationship. That is to say, not all the common gene expression associations between infertility etiologies and other diseases reported by DiseaseConnect web server [5] are translated into a clinical comorbid relationship based on the literature search we have performed. Likewise, Liu et al. [4] found that diseases with shared molecular mechanisms are likely (but not univocally) linked with clinical comorbidity data. In particular, they performed an elegant validation analysis plotting the fraction (y axis) of those mechanism-based disease-disease connections that overlap with disease comorbidity connections of the same disease pairs against various statistical significance (P-value) thresholds (x axis) used to select significant disease–disease connections. The P-value of the connection between two diseases was assessed by a hypergeometric test on shared genes derived from several data sources [GWAS, OMIM, DEG, Gene Reference Into Function (GeneRIF) and disease–gene relationships from literature corpus mining (GeneWays)]. This analysis showed an overall trend for two diseases with stronger mechanism-based connections to be more likely to have a significant clinical comorbidity relationship.

It is important to bear in mind that DiseaseConnect web server [5] is not a static and complete source of information. According to Liu et al. [4], it will be continuously updated because of the rapid accumulation of disease-related omics and text literature data. Thus, the gene-expression data we have extracted from DiseaseConnect web server [5] should not be considered definitive and conclusive.

## Future prospects

The novel approach linking diseases together in a single meta-disease may embrace other non-morbid phenotypic traits. In order for these traits to be included in a given infertility meta-disease, they should share genes with the particular infertility etiology that nucleates that meta-disease and/or at least one disease connected (sharing genes) with that infertility etiology.

Of note, non-morbid phenotypic traits resulting from secondary disturbances induced by a particular infertility condition and/or its associated pathologies cannot be included in this phenomic approach. For instance, PCOS shares genes with “diabetes mellitus, insulin resistant, with acanthosis nigricans” (UMLS ID: C0342278) (Table 2), a condition characterized by insulin resistance and hyperinsulinemia [97]. These metabolic/endocrine disorders may induce PCOS women to manifest cutaneous disarrangements including acanthosis nigricans (sign of insulin resistance) and hirsutism, acne and hair loss (signs of hyperinsulinemia-induced hyperandrogenism) [note that insulin stimulates the ovarian theca cells to secrete androgens and also inhibits hepatic production of sex hormone-binding globulin (SHBG), by this means elevating free testosterone levels (for review, see [52]).

Phenomics is a field of research that is still in its infancy and no clear genetic associations between non-morbid phenotypic traits and infertility etiologies have been yet established. Notwithstanding, several studies have reported significant associations between reproductive and physical/cognitive traits suggesting the presence of phenomic connections between traits. In particular, (1) endometriosis has been related with severe teenage acne [98], leanness, lower body mass index, high sensitivity to sun exposure and pigmentary traits such as natural red hair color, light eyes, nevi and freckles [99-101] (for review, see [102]). (2) Reproductive success [number of living children and (number of living children + (number of living grandchildren)/2)] has been positively correlated with high voice pitch and handgrip strength in indigenous Ovahimba (the last semi-nomadic people of Namibia) women [103]. (3) Sperm concentration, count and motility have been positively correlated with general intelligence (estimated by a *g* factor extracted from 5 well-validated cognitive tests evaluating broad spatial, quantitative and verbal abilities) [104]. And (4) semen quality has been positively correlated with facial attractiveness of men aged 18 to 36 years when facial attractiveness is evaluated by both men and women aged 18 to 25 years in the context of a potential long-term partner [105] (not in the context of a short-term sexual partner evaluated by women [106]).

Further work is needed to untangle the potential genetic, epigenetic and phenotypic relationships that may be present among different infertility etiologies, morbid conditions and physical/cognitive traits.

## Abbreviations

ART: Assisted reproductive technology
ATM: Ataxia-telangiectasia mutated
DEG: Differentially expressed genes
DHEA: Dehydroepiandrosterone
DHEAS: Dehydroepiandrosterone-sulfate
DOR: Diminished ovarian reserve
E_2_: 17β-estradiol
FSH: Follicle stimulating hormone
GeneRIF: Gene Reference Into Function
GnRH: Gonadotropin-releasing hormone
GeneWays: Disease–gene relationships from literature corpus mining
GWAS: Genome-Wide Association
ID: Identification
IGF-I: Insulin-like growth factor I
IVF: In vitro fertilization
KEGG: Kyoto Encyclopedia of Genes and Genomes
LH: Luteinizing hormone
LIF: Leukemia inhibitory factor
LIFR: Leukemia inhibitory factor receptor
OMIM: Online Mendelian Inheritance in Man
PCOS: Polycystic ovary syndrome
PDN: Phenotypic Disease Network
POF: Premature ovarian failure
POI: Primary ovarian insufficiency
SHBG: Sex hormone-binding globulin
UMLS: Unified Medical Language System
